# Analysis of Adult Neurogenesis: Evidence for a Prominent “Non-Neurogenic” DCX-Protein Pool in Rodent Brain

**DOI:** 10.1371/journal.pone.0059269

**Published:** 2013-05-14

**Authors:** Thomas Kremer, Ravi Jagasia, Annika Herrmann, Hugues Matile, Edilio Borroni, Fiona Francis, Hans Georg Kuhn, Christian Czech

**Affiliations:** 1 F. Hoffmann-La Roche AG, Pharma Research & Early Development, DTA Neuroscience, Basel, Switzerland; 2 F. Hoffmann-La Roche AG, pRED Pharma Research and Early Development, Small Molecule Research, Discovery Technologies, Basel, Switzerland; 3 Institut National de la Santé et de la Recherche Médicale UMR-S839, Paris, France; 4 University Pierre et Marie Curie, Paris, France; 5 Institut du Fer à Moulin, Paris, France; 6 Center for Brain Repair and Rehabilitation, Institute for Neuroscience and Physiology, University of Gothenburg, Gothenburg, Sweden; Universidade Federal do ABC, Brazil

## Abstract

Here, we have developed a highly sensitive immunoassay for Dcx to characterize expression in brain and cerebrospinal fluid (CSF) of rodents. We demonstrate that Dcx is widely expressed during development in various brain regions and as well can be detected in cerebrospinal fluid of rats (up to 30 days postnatal). While Dcx protein level decline in adulthood and were detectable in neurogenic regions of the adult rodent brain, similar levels were also detectable in brain regions expected to bear no neurogenesis including the cerebral cortex and CA1/CA3 enriched hippocampus. We monitored DCX protein levels after paradigms to increase or severely decrease adult hippocampal neurogenesis, namely physical activity and cranial radiation, respectively. In both paradigms, Dcx protein- and mRNA-levels clearly reflected changes in neurogenesis in the hippocampus. However, basal Dcx-levels are unaffected in non-neurogenic regions (e.g. CA1/CA3 enriched hippocampus, cortex). These data suggest that there is a substantial “non-neurogenic” pool of Dcx- protein, whose regulation can be uncoupled from adult neurogenesis suggesting caution for the interpretation of such studies.

## Introduction

In the dentate gyrus (DG) of the hippocampus, neurogenesis (NG) occurs constitutively throughout postnatal life in various species including humans [1], [2], [3]. During the last decades, emerging evidence shows that adult hippocampal neurogenesis is implicated in various cognitive and emotional processing abilities but its actual role remains elusive. In rodents, it has been extensively shown that the rate of hippocampal neurogenesis declines with age and is affected by various physiological (enriched environment, physical activity) and pathophysiological conditions (epileptic seizure, stroke, traumatic brain injury). Alterations in adult neurogenesis have been linked to neuropsychiatric diseases, with particular evidence in depression and schizophrenia [4], [5]. Modulation of adult neurogenesis thus presents a novel therapeutic option for various CNS diseases.

The doublecortin gene (Dcx) encodes a microtubule-associated protein which is essential for normal human brain development and mutations cause X-linked lissencephaly [6]. Assessing levels of Dcx has been demonstrated to reflect changes in adult NG and is currently used as a “classical” immunohistochemical marker to detect newborn neurons in brain sections [3], [7]. Dcx starts to be expressed in dividing neuronal precursor cells and persists for approx. 30 days until the cells mature and integrate into the granular cell layer [8]. Dcx has been described as a microtubule stabilizer which can be modulated via its phosphorylation state and has been shown to play an important role in neuronal migration, nuclear translocation and growth cone dynamics [9], [10], [11], [12], [13], [14], [15]. Although studies have shown occasional Dcx-expression in the striatum, corpus callosum or piriform cortex of rodent brain [16], it is generally accepted that Dcx-expression is highly enriched and almost restricted to neurogenic regions. However, recent Dcx immunohistochemical studies in the cerebral cortex of different species such as guinea pig, cat, and primate suggest a broader Dcx expression pattern [17], [18]. Dcx-abundance and localization to certain brain regions varies depending on which Dcx-antibodies have been used [16], [17] and confirmation of Dcx-expression levels with methods other than immunohistochemical stainings (IHC) are missing.

Currently, IHC of different marker proteins are used to quantitatively analyze changes in adult neurogenesis. Albeit changes in cell number and their morphology can be assessed, a quantitative analysis of changes within the hippocampus has several drawbacks, e.g. the procedure is time consuming and susceptible to inaccuracy: sensitivity can vary between different animals as antigenicity is affected by tissue quality and fixation, the signal is amplified non-linearly and signal to background is mostly distinguished by eye. In order to overcome these limitations, we set up a Dcx-immunoassay as a new tool to quantitatively measure Dcx-protein levels in rodent brain tissue.

Our data provide evidence that, in contrast to analysis of Dcx^+^-cells via IHC, total Dcx-protein and mRNA levels are much less affected by changes in neurogenesis. We also show that Dcx expression is much more abundant and not restricted to neurogenic regions within the rodent brain.

## Materials and Methods

### Doublecortin Mesoscale Assays

Sandwich immunoassays were performed using the Meso Scale Discovery assay platform (MSD, Gaithersburg, Maryland, USA) according to the manufactureŕs protocol. In brief, MSD 96-well streptavidin microtitre plates were incubated for 1 h/RT in blocking buffer (50 mM Tris, 60 mM NaCl, 0.1% Tween-20, 5% BSA, pH7.4), washed twice and coated with 25 ul of biotinylated mouse anti-Dcx antibody (mAb49) at a concentration of 10 nM in assay buffer (50 mM Tris, 60 mM NaCl, 1% Tween-20, 0.5% BSA, pH7.4) for 1 h at room temperature. 50 ul of sample diluted in assay buffer and 25 ul of SULFO-tagged mouse anti-Dcx antibody (mAb83) detection antibody at a concentration of 1.5 nM in assay buffer was added and further incubated for 3 h/RT. The plates were washed three times with wash buffer (blocking buffer w/o BSA) and then analyzed after addition of read buffer (MSD) in an MSD Sector Imager 6000 plate reader.

For detection of Dcx in CSF, 10 nM biotinylated rabbit anti-Dcx antibody (ab77450, abcam) was used for capture and 3 nM SULFO-tagged mouse anti-DCX antibody (mAb83) for detection. Recombinant full length Dcx purified from *E.coli* was used as standard.

### Animals

All experiments involving mice and rats were performed by authorized investigators following national and European ethical guidelines.

Dcx knockout mice (deleted for Dcx exon 3) were generated by using the Cre-loxP site-specific recombination system, and crossed onto the C57BL/6N background as described previously [19]. Dcx is present on the X chromosome, so male hemizygote mice have no functional Dcx protein. For analyses male hemizygote knockout mice were compared with littermate male wild type mice. These were generated by crossing heterozygote females with pure C57BL/6N males (Charles River, France). Mice were genotyped by PCR as described previously [19]. Adult mice were sacrificed by anesthesia and decapitation. Brains were removed from skulls and individual hemispheres snap frozen in liquid nitrogen.

### Rat Irradiation Experiments

For irradiation, a linear accelerator (Varian Clinac 600CD) with 4 MV nominal photon energy and a dose rate of 2.3 Gy/minute was used. A single dose of 6 Gy or 12 Gy was administered to each animal. The dose variation within the target volume was estimated to be ±5%. Ten-day-old rats were anesthetized with an intraperitoneal (i.p.) injection of tribromoethanol (Avertin; Sigma-Aldrich, Stockholm, Sweden, http://www.sigmaaldrich.com) and placed in a prone position (head to gantry) on an expanded polystyrene bed. The whole brain, including the olfactory bulbs, was irradiated with a radiation field of 2×2 cm. The source to skin distance was approximately 99.5 cm. To spread the dose evenly throughout the tissue, the head was covered with a 1-cm-thick tissue-equivalent material. The entire procedure was completed within 10 minutes. After irradiation, the pups were returned to the dams until weaning and sacrificed 7-weeks after irradiation. The sham-irradiated control animals were anesthetized but not subjected to irradiation.

### Running Wheel Experiment

In two sets of experiments, female mice of approximately 2 months of age were randomly divided into two groups. The control group was placed in standard housing conditions (three animals per cage). The running group was housed in a rat cage (three animals per cage) with free access to running wheels (Sandown Scientific). Animals were anaesthetized after two weeks using isofluorane and killed by de-capitation. For protein analysis, C57BL/6N mice (Charles River, France, N = 12/group) were used while for RNA analysis, Sv129Ev (Taconic, Denmark, N = 15/group) were used.

### Tissue Processing

Unless otherwise noted, brains with intact olfactory bulb were removed from the skull and split into its hemispheres. Hemispheres were either directly immersion-fixed in formaldehyde solution or further dissected to prepare respective brain regions. Tissue was either placed in RNAlater solution (Ambion) or snap-frozen on dry ice for subsequent RNA- or protein-analysis.

For protein analysis, tissue was homogenized in 10 Vol of modified RIPA-buffer (50 mM Tris-HCl, pH 7.4; 1% NP-40; 0.25% Na-deoxycholate; 150 mM NaCl, 1 mM EDTA) using PreCellys®-24 Tissue homogenizer. Insoluble material was pelleted by centrifugation at 10.000 g for 10 Min at 4°C. Total protein content of the resulting supernatant was determined using Biorad DC protein assay. Samples were appropriately diluted in mesoscale assay buffer for measuring DCX-protein levels. For detection of Dcx in CSF during rat development, CSF samples and corresponding brain tissue samples at different developmental stages (starting from postnatal day 5 (P5), P10, P20, P30 and P40; N = 4) from Spraque-Dawley rats were purchased from JSW Lifesciences (Austria).

### Immunohistochemistry

Unless otherwise noted, brains with intact olfactory bulb were removed from the skull and longitudinally split into halves. Right hemispheres were fixed for 24 hrs in 10% formalin. Coronal sections of equivalent regions of the caudal and rostral hippocampus were embedded in paraffin with 3 equivalent regions of runners and controls each per block. From blocks with caudal hippocampus consecutive slides were stained with anti-DCX (rabbit polyclonal, Abcam) primary antibody on Ventana Discovery® XT autostainer. Four series per block were produced with a leave-out of 10 slides in between.

### Dcx protein Expression Analysis (Western Blot)

Anti-Dcx (rabbit polyclonal, Abcam®) was used as primary antibody. Detection of bound primary antibody was performed using peroxidase-conjugated donkey anti-rabbit 1∶10.000 and ECL substrate according to the manufacturer’s protocol (Amersham Bioscience). Signals were recorded on Hyperfilm ECL chemiluminescence films (Amersham Bioscience).

### Image Analysis

All slides were scanned with Aperio ScanScope®. Semi-automated image-analysis of immunostaining was performed with Definiens Tissue Studio™ software on the basis of manually annotated regions of interest comprising the dentate gyrus. Concomitantly semi-quantitative grading of DCX staining was performed by a pathologist.

### RNA Preparations and RT-PCR Analysis

Total RNA from snap-frozen brain tissue was isolated using the Norgen All-in-One Purification Kit (Norgen, Canada) according to the manufacturer’s protocol with slight modifications. In brief, tissue was homogenized with TissueLyserII (Qiagen) and total RNA purified. RNA was DNase digested using DNaseI (Roche), re-purified using RNeasy MinElute (Qiagen) and quantified by photometry. RNA integrity was verified by gel electrophoresis. 4 ng total RNA was used for RT-PCR in a 384-well plate on a Roche Light Cycler LC480. The following taqman assays from ABI were used: Rn01775763_g1 for rat glyceraldehyde-3-phosphate dehydrogenase (GAPDH), Rn00584505_m1 for rat doublecortin (Dcx). A relative quantification method was based on the expression of the rat GAPDH gene.

For RNA analysis of mouse tissue, relative transcript expression was assessed with a Fluidigm Biomark Dynamic Array by using the following taqman assays from ABI: Mm00438401_m1 for mouse DCX, Mm03053654_s1 for mouse Sox11, Mm01351985_m1 for mouse Tbr2/EOMES. Relative quantification of RNA between dentate gyrus and CA1/CA3-enriched hippocampus was based on the expression of the following housekeeping genes: Mm00437762_m1 for B2 M, Mm01197698_m1 for HMBS, Mm01143545_m1 for GUSB, Mm01352366_m1 for SDHA and Mm00446973_m1 for TBP.

## Results

### Dcx Protein-levels in Tissue Homogenates and CSF during Development

**Figure 1:**
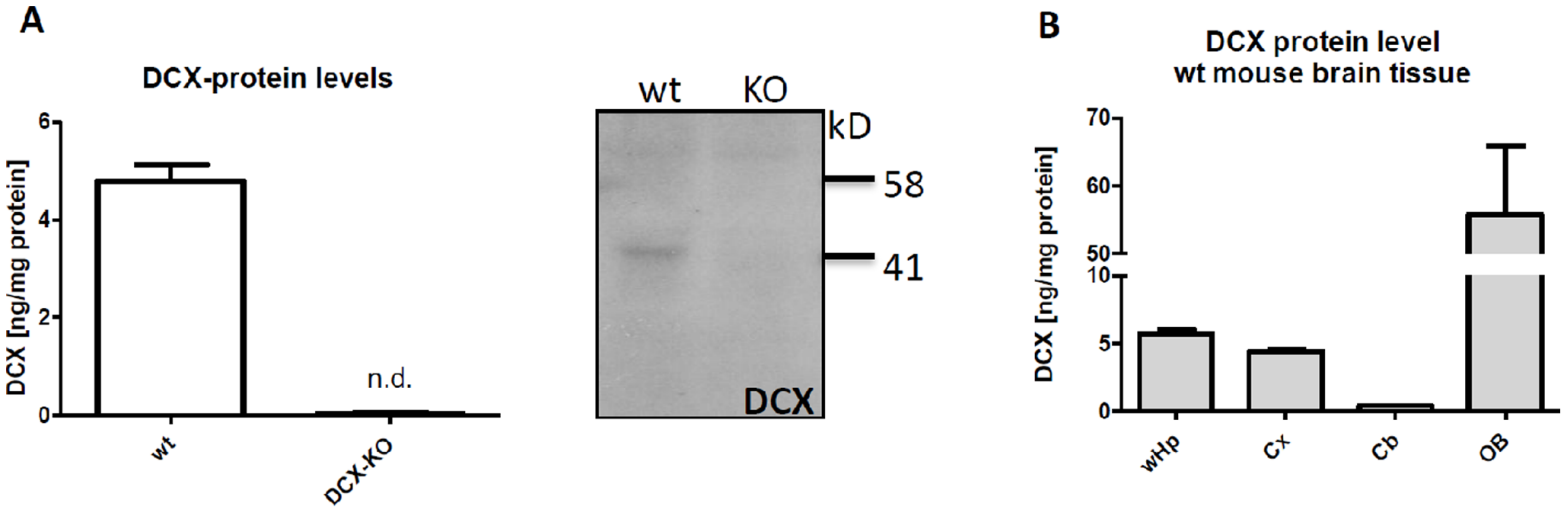
Murine Dcx-protein expression using a Dcx-specific immunoassay. A, Left, Dcx is detected in whole brain homogenates from adult C57BL/6 wt and Dcx-KO mice using a sandwich immunoassay. No signal can be observed in Dcx-KO mice (N = 3). Right, Representative Dcx immunoblot. Dcx can be detected in hippocampal tissue of adult wildtype but not Dcx KO mice. B, expression levels of Dcx-protein in various mouse brain regions (wHp: whole hippocampus, Cx: cortex, Cb: Cerebellum, OB: olfactory bulb, N = 6).

For quantitative measurements of human and rodent Dcx, we generated highly sensitive sandwich immunoassays which enabled detection of Dcx protein in tissue and body fluids at concentrations in the range up to low picograms per milliliter (Fig. S1). Cross-species reactivity to mouse, rat and human Dcx was likely due to high sequence similarity and was confirmed by epitope mapping of antibodies (data not shown). As Dcx also shows high homology to doublecortin-like kinase isoforms, we used adult Dcx-KO mice to validate our immunoassay for specificity. Dcx-protein can be detected in brain homogenates of adult wild type littermate mice while Dcx could not be detected in KO-tissue (Fig. 1a).

We next analyzed Dcx protein levels in different regions from adult mouse brain. As shown previously, Dcx is highly expressed in the olfactory bulb; moderate expression is observed in whole hippocampus. Intriguingly, analysis of cortical tissue using this immunoassay reveals moderate expression of DCX-protein almost comparable to hippocampal protein levels. Furthermore, Dcx-protein can be also detected in the cerebellum albeit at low levels (Fig. 1b).

**Figure 2:**
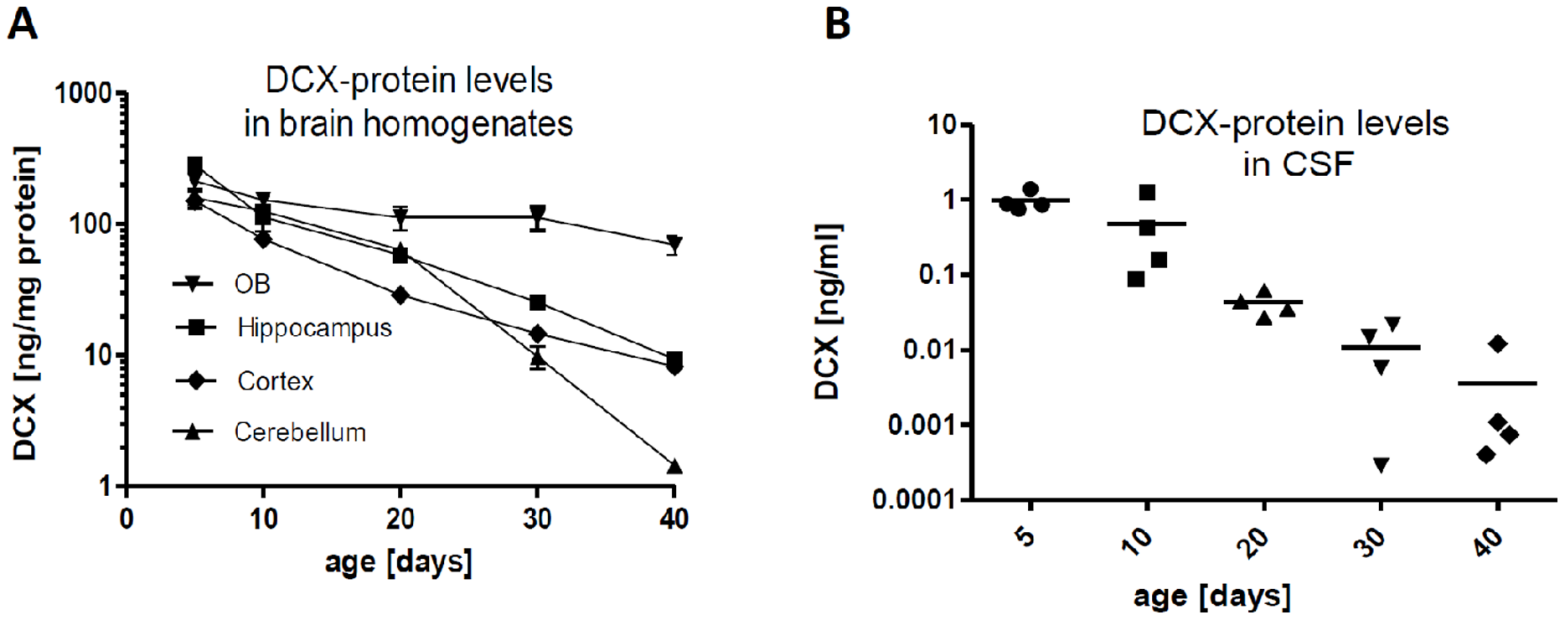
Dcx protein-levels in rat brain homogenates and CSF during development. Olfactory bulb, whole hippocampus, pieces of cerebral cortex and cerebellum, and CSF were analyzed from rats at different developmental stages (postnatal day 5 to 40). A, Dcx-protein levels in rat brain tissue homogenates during postnatal development. B, Dcx-protein levels in CSF during postnatal development (N = 4).

Dcx is used as immunohistochemical marker for neuronal precursor cells and immature neurons [8] and has been shown to be highly expressed during neurodevelopment with peak expression around birth and constant decline during postnatal development [20]. Interestingly, proteomic analyses of CSF obtained from human embryos show that Dcx can be detected in this fluid [21]. We analyzed rats at different stages of development starting from postnatal day 5 to 40 in order to test for occurrence of Dcx-protein levels in rodent CSF and respective brain tissues. In accordance with previous findings in mouse brain, Dcx shows highest expression at P5 and declines with postnatal development [7]. At P40, Dcx shows a similar tissue expression pattern as observed for adult mouse brain with comparable levels in hippocampal and cortical tissue (Fig. 2a). Immunoprecipitation of Dcx from cortical tissue followed by mass spectrometric analysis confirmed presence of Dcx protein in rat cortex (data not shown).

Analysis of rat CSF shows that Dcx can be detected in CSF during postnatal development. Highest levels of DCX can be detected in P5 rats, there is a decline with age (P5∶975±144 pg/ml; P10∶481±269 pg/ml; P20∶42±8 pg/ml; P30∶10±4 pg/ml) and CSF-Dcx is below the detection limit in 3 out of 4 animals at P40 (Fig. 2b). Additional analysis of 50 CSF samples of adult rat samples showed no Dcx protein expression (data not shown).

### Dcx-mRNA and Protein upon Changes in Neurogenesis - Cranial Irradiation (rat)

Immunohistochemical studies predict a strong Dcx-expression in the neurogenic regions of the brain, which are the subgranular zone (SGZ) of the hippocampus and the subventricular zone (SVZ). From the SVZ, Dcx^+^-cells migrate along the rostral migratory stream (RMS) to the olfactory bulb where a high density of Dcx^+^-immunoreactive (IR) cells is observed [8].

To test whether Dcx-protein levels reflect changes in adult neurogenesis, we compared Wistar rats subjected to cranial irradiation at P10 with sham treated animals. Cranial irradiation results in a robust, long-lasting and almost complete loss of neurogenic pools in both SVZ and SGZ and results in complete loss of Dcx-immunoreactivity in hippocampal and olfactory bulb tissue [22]. Animals were sacrificed 7 weeks after irradiation and the above mentioned tissue types were dissected from each hemisphere and used for either protein- or mRNA- expression analysis.

**Figure 3:**
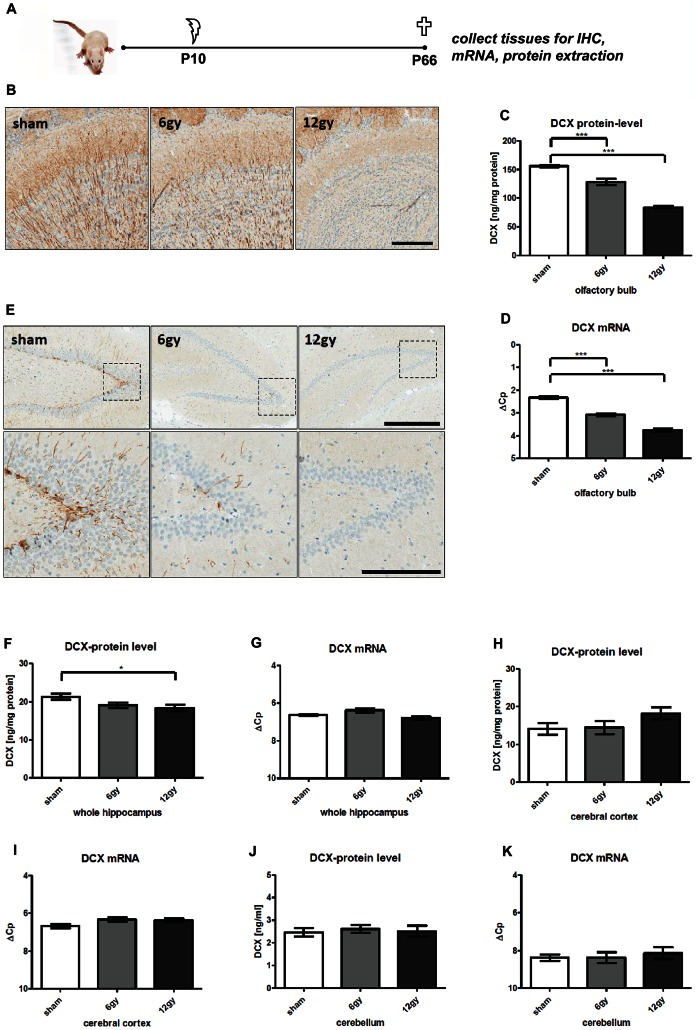
Irradiation-induced ablation of neurogenesis. A, schematic diagram of experimental procedures. Female wistar rats received a high (12 gy) or low (6 gy) irradiation dose or were sham-treated at P10 (N = 10 per group). Mice were sacrificed 7 weeks after treatment. A subset of brains were processed for immunohistochemistry (12 gy: N = 2, 6 gy: N = 3, sham: N = 3). Residual brains were split into hemispheres and dissected for mRNA and protein analysis (N = 6/group). B, Representative images of Dcx-IR in the olfactory bulb of formalin-fixed paraffin embedded (FFPE) sections. Left-to-right: A high density of dendritic Dcx-IR is observed in the olfactory bulb granular layer sham-irradiated animals. Dendritic labeling is reduced with low-irradiation and virtually absent in animals after 12 gy-irradiation. Scale bar: 200 µm. C, bar graphs of Dcx-protein-levels in the olfactory bulb. A dose-dependent decrease in Dcx-protein levels is observed in irradiated animals vs sham-controls. D, bar graphs of Dcx-mRNA-levels in the olfactory bulb. A dose-dependent decrease in Dcx-mRNA levels is observed in irradiated animals vs sham-controls. E, Representative images Dcx-IR in the dentate gyrus of FFPE sections. Upper panel: overview of Dcx-IR in the dentate gyrus. Left-to-right: Dcx-IR is restricted to cells in the dentate gyrus SGZ with dendrites spanning into the granular and molecular layer. Lower panel: higher magnification of the SGZ. F, bar graphs of Dcx-protein-levels in the hippocampus. A slight dose-dependent decrease in DCX-protein levels is observed in irradiated animals. G, bar graphs of Dcx-mRNA-levels in the hippocampus. DCX-mRNA levels do not change significantly between sham and irradiated groups. H, bar graphs of Dcx-protein-levels in the cerebral cortex. I, bar graphs Dcx-mRNA-levels in cerebral cortex. DCX-mRNA levels do not change significantly between sham and irradiated groups. J, bar graphs of Dcx-protein-levels in the cerebellum. K, bar graphs of Dcx-mRNA-levels in cerebellum. Dcx-mRNA levels do not change significantly between sham and irradiated groups. Dunnett’s Multiple Comparisons Test.

As previously described [8], we detect a strong dendritic Dcx-IR in the granular layer of the olfactory bulb in sham treatment animals (Fig. 3). Dcx-IR is strikingly reduced in 6 gy-treated animals while apparently no dendritic Dcx-IR is detected in the granular layer of 12 gy-treated animals (Fig. 3b). Accordingly, Dcx-protein levels in the olfactory bulb are highest in sham-treated animals and show a dose-dependent decrease with irradiation (sham: 155.8±4.7 ng/mg; 6 gy: 125.8±5.7 ng/mg; 12 gy: 83.8±13.4 ng/mg) (Fig. 3c). Of note, Dcx-protein levels upon high dose irradiation still consist of approx. 50% protein compared to sham-treated animals. Similarly, Dcx-mRNA levels in the olfactory bulb are highest in sham-treated animals (2.33±0.16 ΔCp) and decrease dose-dependently with irradiation (6 gy: 3.08±0.16 ΔCp; 12 gy: 3.77±0.18 ΔCp) (Fig. 3d).

For the hippocampus, we observed strong dendritic Dcx-IR in the SGZ of the DG (Fig. 3e) in sham treatment animals. No Dcx-IR could be detected in other regions of the hippocampus. Dcx-IR is strikingly reduced in 6 Gy-treated animals while no Dcx-IR is detectable in 12 gy-treated animals (Fig. 3e). However, Dcx-protein levels in the hippocampus only slightly change towards lower levels and reach statistical significance only in the high-dose group (sham: 21.3±2.2 ng/mg; 6 gy: 19.2±1.5 ng/mg; 12 Gy: 18.4±2.0 ng/mg) (Fig. 3f). Dcx-mRNA levels do not change significantly between sham and irradiated groups (sham: 6.6±0.1 ΔCp; 6 gy: 6.4±0.3 ΔCp; 12 gy: 6.8±0.8 ΔCp) (Fig. 3g). Analysis of cortical and cerebellar tissue revealed that Dcx-protein and mRNA- levels were not affected by cranial irradiation (Fig 3H–K Cortex protein: sham: 14.11±1.5 ng/mg; 6 gy: 14.5±1.7 ng/mg; 12 gy: 18.42±1.6 ng/mg. Cortex mRNA: sham: 6.7±0.1 ΔCp; 6 gy: 6.3±0.1 ΔCp; 12 gy: 6.4±0.1 ΔCp. Cerebellum protein: sham: 2.5±0.19 ng/mg; 6 gy: 2.6±0.17 ng/mg; 12 gy: 2.5±0.24 ng/mg. Cerebellum mRNA: sham: 8.4±0.1 ΔCp; 6 gy: 8.4±0.1 ΔCp; 12 gy: 8.1±0.1 ΔCp).

Our results therefore strongly suggest a broader DCX-expression consisting of a “neurogenic” and “non-neurogenic” pool, the latter not being affected by cranial irradiation.

### Dcx-mRNA and Protein in the Hippocampus - Dcx^+^-cells in the SGZ Comprise a Subfraction of Whole Hippocampal Dcx Protein Expression

Based on the results of the irradiation experiments, we speculated that changes in Dcx-mRNA and protein levels only reflect changes in neurogenesis when a sufficiently high density of “neurogenic” Dcx^+^-cells are present that are readily detectable by immunohistochemistry of the analyzed tissue (e.g. olfactory bulb). Based on immunohistochemistry, Dcx-protein expression is believed to be restricted to the SGZ of the dentate gyrus in adult brain. In a new set of experiments, we performed a 14-day voluntary running wheel experiment in mice. This enabled us to measure Dcx protein levels in a neurogenesis induction paradigm and to exclude that our results in the irradiation experiment were due to the irradiation procedure. For this analysis, we separated whole hippocampal tissue into dentate gyrus and CA1/CA3 enriched residual hippocampus (resHp) according to Hagihara and colleagues to directly discriminate between non-neurogenic and neurogenic regions within the hippocampus and to compare both DCX-mRNA and protein-levels in extracts with Dcx-IHC [23]. Analysis of genes known to be enriched in dentate gyrus (*Tdo2*) or the CA1/CA3-region (*Stmn2*) by RT-PCR confirmed our dissection procedure (data not shown).

**Figure 4:**
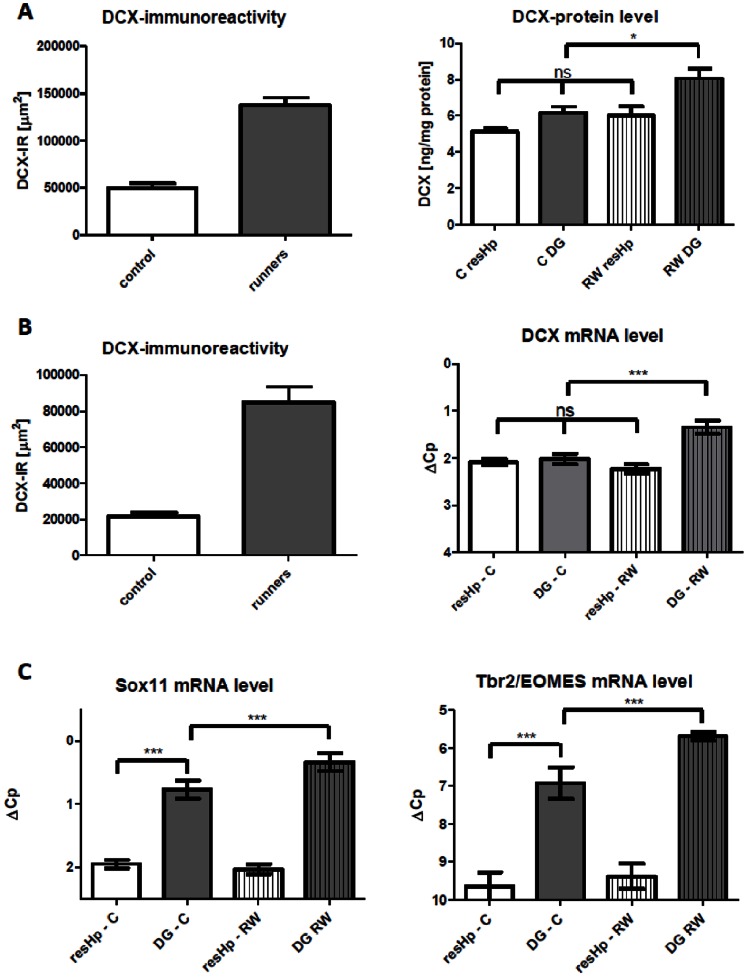
14-day voluntary running wheel experiment. In two separate experiments, adult mice with or without access to a running wheel were sacrificed after 2 weeks. The right hemisphere was dissected for either protein or mRNA-analysis while the left hemisphere was used to confirm exercise-induced increase in Dcx-IR via immunohistochemistry. A, Dcx-IR was quantified by calculating the total Dcx-IR area in µm^2^ for four different sections within the dorsal hippocampus (left). Bar graph of hippocampal Dcx-protein-levels in DG and resHp (right). N = 12/group. B, Dcx-IR was quantified by calculating the total Dcx-IR area in µm^2^ for four different sections within the dorsal hippocampus (left). Bar graph of hippocampal Dcx-mRNA-level in DG and resHp (right). N = 15/group. C, Bar graph mRNA-level in DG and resHp for Sox11 and Tbr2/EOMES. N = 15/group. Bonferroni’s Multiple Comparisons Test.

The results support our previous findings of a non-neurogenic pool of Dcx. In both groups of animals Dcx protein could be detected in the non-neurogenic CA1/CA3 enriched hippocampus at comparable levels (control group: 5.1±0.2 ng/mg vs. runners: 6.0±0.5 ng/mg). Running wheel treatment leads to a significant increase in Dcx protein levels in the DG (control: 6.1±0.3 ng/mg, runners: 8.1±0.5 ng/mg) (Fig. 4a). Similarly, Dcx-mRNA levels do not differ between resHp and DG in the control group (controls: resHp 2.09±0.07 ΔCp; DG 2.02±0.11 ΔCp) but specifically increase in the dentate gyrus upon running wheel (runners resHp 2.28±0.09 ΔCp, DG 1.35±0.14 ΔCp) (Fig. 4b). We next compared DG and resHp mRNA-levels of two other neurogenic markers, Tbr2/EOMES and Sox11, the latter showing an almost complete overlap with Dcx in IHC staining [24], [25]. Both markers show a significant enrichment in the DG which increases with running (Sox11: Control: resHp: 1.95±0.07 ΔCp, DG: 0.77±0.15 ΔCp. Runner: resHp: 2.03±0.08 ΔCp, DG: 0.34±0.14 ΔCp. Tbr2: Control resHp: 9.65±0.37 ΔCp; DG: 6.92±0.42 ΔCp. Runner: resHp: 9.38±0.33 ΔCp; DG: 5.69±0.10 ΔCp (Fig. 4c), and a lower basal expression in the rest of the hippocampus.

These results demonstrate that Dcx protein is highly expressed in during development but persists at substantial basal levels in the rodent brain. Upon induction of neurogenesis, total Dcx mRNA and protein expression levels are induced in the dentate gyrus, most likely by an increased number in migrating neuroblasts and immature neurons.

## Discussion

Here, we used a sensitive Dcx-immunoassay to demonstrate that Dcx protein expression is more abundant in rodent brain than would have been predicted from previous studies based on immunohistochemical data. Dcx protein expression is not restricted to neurogenic regions but can be detected in substantial amounts in other areas of the rodent brain. Using irradiation-induced ablation of neurogenesis and physical exercise, we show that Dcx-immunoreactive cells of the dentate gyrus subgranular zone bear only a fraction of the total hippocampal Dcx protein pool. Dcx protein expression thus only partially reflects neurogenesis which depends on the overall fraction of “neurogenic” Dcx-immunoreactive cells within the respective tissue.

Our results are in contrast to results obtained using transgenic mouse models in which the Dcx promoter was used to drive expression of fluorescent reporter proteins [4]. A plausible explanation could be that reporter protein expression under the promoter of a gene of interest might not reproduce perfectly the corresponding gene expression pattern as differences in stability of the mRNA and protein can significantly vary between reporter and endogenous protein (for review, see [26]). In nestin–EGFP transgenic mice, a longer persistence of the reporter protein EGFP compared to endogenous nestin protein has been postulated to be the cause of the increased stability of the EGFP [27]. Vice versa, expression of a highly stable mRNA or protein can be underestimated in fluorescent reporter mice. In line with this hypothesis, Dcx–GFP transgenic mice from the Gene Expression Nervous System Atlas BAC transgenic (GENSAT) project (www.gensat.org) [28], in which the EGFP reporter gene is inserted immediately upstream of the Dcx coding sequence, show a much broader EGFP signal expression in adult brain which is in line with our findings. Since we can also observe broad Dcx mRNA expression, stability might at least partially result from a stable mRNA. Of note, a striking feature of Dcx mRNA is a 7.9 kb long 3′ untranslated region [6] containing AU-rich regulatory elements (which is also preserved in the GENSAT Dcx-BAC mice) which could point towards post-transcriptional regulatory mechanisms.

The *doublecortin* (*Dcx*) gene, has 2 close paralogs, *doublecortin-like kinase* 1 and 2 (*Dclk* 1 and 2) which partially compensate for Dcx in Dcx KO mice [13], [29], [30] and potential cross-reactivity to these paralogs could explain the broad expression pattern observed using our DCX immunoassay. Although recent evidence points towards to some cross-reactivity of one of our antibodies (ab77450, abcam) with the alternative Dclk11 splice variant doublecortin-like (DCL) in western blots [31], [32], we observe no residual signal in Dcx KO mice, which have been shown to have no concomitant reduction of these paralogs, including DCL, at both RNA and protein levels [33]. Another possibility for the discrepancy observed between immunohistochemical and biochemical analysis of Dcx could be differential detection of the epitope in solution compared to fixed tissue. Microtubule-affinity of Dcx protein is regulated by its phosphorylation status which could also affect antibody-epitope interaction [14], [15], [34], [35]. Antibodies used in our study were generated against an unphosphorylated Dcx peptide or protein, respectively, so phosphorylated Dcx may not be detectable with these antibodies. However, immunohistochemical analyses of Dcx in rodent brain have been performed using various antibodies including those directed against the N-terminus of Dcx [36], [37] and so far, considerable detection of Dcx immunoreactivity in cerebral cortex of rodent brain has not been described. As our immunoassay utilizes an antibody directed against the C-terminus which we and others have been used for immunohistochemical studies [37], [38], [39], we believe that selective detection of certain Dcx epitopes via our immunoassay compared to immunohistochemistry seems unlikely.

Our results show that a sensitive detection method is crucial to understand the full role of Dcx in the brain. While immunohistochemical analysis enables cellular and subcellular localization of the protein of interest, it cannot be used for quantitative measurements mainly due to differential accessibility of the antigen(s) and non-linear amplification of the signal. Immunoassay-based detection enables quantitative and highly sensitive protein measurements, however, without cellular information in complex tissue samples. Combining both methods, we provide evidence that “non-neurogenic” Dcx is expressed by other cells, most likely at comparably low levels that are below detection limit by immunohistochemical analysis. Although we cannot fully exclude that a small fraction of the Dcx protein in non-neurogenic regions derives from neurogenic cells, we clearly demonstrate that overall Dcx levels are unaffected by stimuli that have an impact on neurogenic cell populations (running wheel, irradiation). Dcx mRNA expression in cells of the CA1/CA3 region and cortex of adult mouse brain is further supported by Allen brain atlas in-situ hybridization data with Dcx-specific probes in adult mouse brain (data available from http://mouse.brain-map.org/). Interestingly, previous immunohistochemical studies in rodent brain have shown that small subpopulations of Dcx-positive cells can be detected in other regions than neurogenic niches such as the corpus callosum, the piriform cortex layer II, striatum and even cerebellum [16], [40]. Dcx^+^-cortical cells have been either classified as mature neurons undergoing structural plasticity [16] or defined as neurons in a prolonged immature state [41]. Currently, it is still under debate whether these cells have pre- [41] or postnatal origin [42], [43], [44]. Studies in adult guinea pigs, rabbits and primates show that Dcx^+^- cells can be detected additionally throughout the neocortex [45], [46]. Dcx^+^-cells have been detected throughout the different cortical layers comprising up to ∼5% of all cortical cells in cynomolgus monkeys [17], while a recent study in middle-aged marmosets revealed an unexpectedly high number of Dcx^+^-cells in the amygdala [47].

Altogether, these studies indicate that in addition to its neurodevelopmental role, Dcx might also play a role in adult neuronal plasticity and migration. An increase in Dcx^+^-cells with a broad distribution in the neocortex of higher mammalian species was postulated to be an evolutionary adaption to increased brain size in order to retain or increase structural plasticity and interconnectivity [17] and suggests an additional role for Dcx particularly for highly developed species. Our data might suggest that Dcx is present in such cells in low levels even in rodents.

Mutations in doublecortin cause severe cortical malformations (doublecortex syndrome/lissencephaly) associated with intellectual disability and drug-resistant epilepsy. These clinical consequences appearing generally early in childhood, are believed to be due to the disorganization of the cortex and presence of many aberrantly positioned ‘heterotopic’ neurons in the white matter. Our findings indicating the presence of immunoreactive doublecortin in non-neurogenic regions of the adult mouse brain may suggest a function for this protein in mature nervous system cells. Mutations in doublecortin in human could potentially perturb such a function, which may hence also potentially contribute to these disorders in adulthood. Further work questioning Dcx’s adult functions besides its neurodevelopmental role may shed light on this question [48], [49]. Specific deletion of Dcx, e.g. in postnatal forebrain, in conditional Dcx knockout mice could address a potential role in adult cortex and to assess specific consequences of its loss of function in mature nervous system cells. Dcx co-precipitates with adapter proteins involved in protein sorting and vesicular trafficking [50]. Subcellular localization in hippocampal neuron cultures revealed that Dcx shows high enrichment with microtubules extending into the actin-rich lamellar regions and facilitate axonal growth and collateral branching and its localization and function is determined by its phosphorylation state [14], [15], [51]. We speculate that Dcx-protein levels reflect high motility and/or structural plasticity of a given cell or a given brain region. Differences in Dcx-levels in different brain regions could therefore mirror differences in their capability to undergo structural plasticity. We observed a constant decline of Dcx-protein levels with age in mice in all brain regions analyzed (data not shown) which could in turn reflect reduced plasticity of inter-neuronal connections with age (in line with [52], [53]). These findings are consistent with recent literature data that strongly favor an important role for Dcx besides neurogenesis in the adult brain. As we did not observe changes of Dcx-expression in non-neurogenic regions in the current study, it remains to be determined whether the basal Dcx-levels in cells besides immature neurons can be dynamically regulated.

Using our immunoassay, we show that Dcx-protein levels are also detectable in rat CSF during postnatal development. DCX protein has been postulated as a potential CSF biomarker to measure both severity and neurologic outcome in childhood traumatic brain injury (TBI) [54]. Our immunoassay enables enhanced sensitivity and accuracy of Dcx-detection in CSF which will help to prove its utility as prognostic biomarker.

In summary, our newly developed immunoassay enables precise and quantitative measurements of Dcx in tissue and body fluids and will help to unveil yet undiscovered functions for Dcx in particular for higher species.

## Supporting Information

Figure S1 **Characteristics of the Dcx immunoassay.** A, Calibration curve of the DCX immunoassay using purified recombinant human Dcx protein as standard. B, Analytical linearity of Dcx protein concentrations in adult mouse brain tissue extracted in RIPA buffer. C, Signal recovery of recombinant human Dcx protein spiked in adult rat CSF.
(TIF)
