# Supplementary figures and images for: Analysis of Adult Neurogenesis: Evidence for a Prominent “Non-Neurogenic” DCX-Protein Pool in Rodent Brain
Source: PLoS One. 2013 May 14;8(5):e59269. doi: 10.1371/journal.pone.0059269 (PMC3653925; doi:10.1371/journal.pone.0059269)

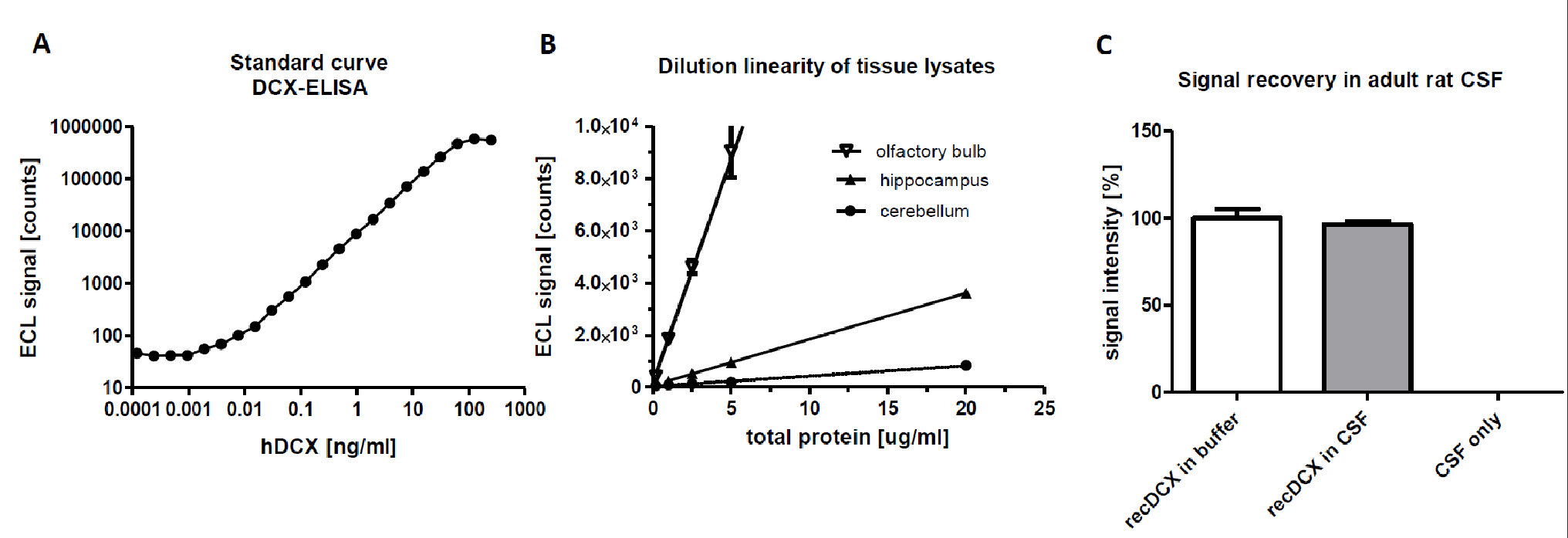

Supplement: Figure S1 — Characteristics of the Dcx immunoassay. A, Calibration curve of the DCX immunoassay using purified recombinant human Dcx protein as standard. B, Analytical linearity of Dcx protein concentrations in adult mouse brain tissue extracted in RIPA buffer. C, Signal recovery of recombinant human Dcx protein spiked in adult rat CSF. (TIF) [file pone.0059269.s001.tif]
